# Design and rationale of the EMPA‐VISION trial: investigating the metabolic effects of empagliflozin in patients with heart failure

**DOI:** 10.1002/ehf2.13406

**Published:** 2021-05-06

**Authors:** Moritz J. Hundertmark, Olorunsola F. Agbaje, Ruth Coleman, Jyothis T. George, Rolf Grempler, Rury R. Holman, Hanan Lamlum, Jisoo Lee, Joanne E. Milton, Heiko G. Niessen, Oliver Rider, Christopher T. Rodgers, Ladislav Valkovič, Eleanor Wicks, Masliza Mahmod, Stefan Neubauer

**Affiliations:** ^1^ Oxford Centre for Clinical Magnetic Resonance Research (OCMR), Division of Cardiovascular Medicine, Radcliffe Department of Medicine University of Oxford, John Radcliffe Hospital Headley Way Oxford OX3 9DU UK; ^2^ Diabetes Trials Unit, Oxford Centre for Diabetes, Endocrinology and Metabolism, Radcliffe Department of Medicine University of Oxford Oxford UK; ^3^ Boehringer Ingelheim Pharma GmbH & Co. KG Biberach an der Riss Germany; ^4^ Boehringer Ingelheim International GmBH Ingelheim Germany; ^5^ Oxford NIHR Biomedical Research Centre Oxford University Hospitals Oxford UK; ^6^ Wolfson Brain Imaging Centre, Department of Clinical Neurosciences Cambridge Biomedical Campus Cambridge UK; ^7^ Department of Imaging Methods, Institute of Measurement Science Slovak Academy of Sciences Bratislava Slovakia; ^8^ John Radcliffe Hospital Oxford University Hospitals NHS Foundation Trust Oxford UK

**Keywords:** Heart failure, Diabetes, SGLT2 inhibitors, ^31^P‐MRS, Trial design, Empagliflozin

## Abstract

**Aims:**

Despite substantial improvements over the last three decades, heart failure (HF) remains associated with a poor prognosis. The sodium‐glucose co‐transporter‐2 inhibitor empagliflozin demonstrated significant reductions of HF hospitalization in patients with HF independent of the presence or absence of type 2 diabetes mellitus in the *EMPEROR‐Reduced* trial and cardiovascular mortality in the *EMPA‐REG OUTCOME* trial. To further elucidate the mechanisms behind these positive outcomes, this study aims to determine the effects of empagliflozin treatment on cardiac energy metabolism and physiology using magnetic resonance spectroscopy (MRS) and cardiovascular magnetic resonance (CMR).

**Methods and results:**

The *EMPA‐VISION* trial is a double‐blind, randomized, placebo‐controlled, mechanistic study. A maximum of 86 patients with HF with reduced ejection fraction (*n =* 43, Cohort A) or preserved ejection fraction (*n =* 43, Cohort B), with or without type 2 diabetes mellitus, will be enrolled. Participants will be randomized 1:1 to receive either 10 mg of empagliflozin or placebo for 12 weeks. Eligible patients will undergo cardiovascular magnetic resonance, resting and dobutamine stress MRS, echocardiograms, cardiopulmonary exercise tests, serum metabolomics, and quality of life questionnaires at baseline and after 12 weeks. The primary endpoint will be the change in resting phosphocreatine‐to‐adenosine triphosphate ratio, as measured by ^31^Phosphorus‐MRS.

**Conclusions:**

*EMPA‐VISION* is the first clinical trial assessing the effects of empagliflozin treatment on cardiac energy metabolism in human subjects *in vivo*. The results will shed light on the mechanistic action of empagliflozin in patients with HF and help to explain the results of the safety and efficacy outcome trials (*EMPEROR‐Reduced and EMPEROR‐Preserved*).

## Introduction

Chronic heart failure (HF) is a progressive syndrome caused by an imbalance of oxygen supply provided by the heart and metabolic demand by various tissues. Its symptoms and multi‐organ adverse effects lead to high morbidity, poor quality of life (QoL), poor survival rates, and significant economic strain on health care systems worldwide.^1^, ^2^ With estimates of over 60 million people affected globally, this epidemic is predicted to substantially increase over the next decade.^3^ The global socio‐economic burden of HF comprises an annual cost of $108 billion and accounts for approximately 2% of total health care expenditure.^4^

Heart failure phenotyping is based on echocardiographic measurement of left ventricular ejection fraction (LVEF) and categorizes the syndrome into two main groups: HF with reduced ejection fraction (HFrEF; LVEF < 40%) and preserved ejection fraction (HFpEF; LVEF ≥ 50%).^2^ HFrEF and HFpEF have approximately equal prevalence and 5 year mortality rates.^5^ HFpEF is more prevalent in older, female patients and shows a steep increase with advancing age, whereas HFrEF plateaus with advancing age and is more frequently seen in male patients.^6^

In HFrEF, morbidity and mortality remain high despite the success of therapeutic interventions.^7^ Given the ageing population, the number of patients with HFpEF is constantly rising and frequency of hospital admissions is comparable with those with HFrEF.^8^ Even more concerning, the mainstay of treatment for HFpEF is symptom relief, while no outcome‐modifying treatment is available, a fact underscoring the unmet therapeutic need in this group.

Up to 40% of patients with HF have concomitant type 2 diabetes mellitus (T2DM), and a similar proportion presents with impaired glucose tolerance, often termed ‘pre‐diabetes’, both of which increase mortality.^9^ Interestingly, even in the absence of diabetes, insulin sensitivity decreases as HF progresses, indicating a possible link between HF and gluco‐metabolic disturbances.^10^

Empagliflozin is a selective sodium‐glucose co‐transporter‐2 inhibitor (SGLT2‐i) licensed for treating patients with T2DM by promoting urinary glucose excretion.^11^ In *EMPA‐REG OUTCOME*, treatment with empagliflozin reduced the risk of death from cardiovascular (CV) causes by 38% and HF hospitalization by 35%, in patients with T2DM and established CV disease.^12^ In the trial, the reduction in the composite primary outcome (3‐point major adverse CV events) was specifically driven by a reduction of endpoints associated with the progression of HF.

Dedicated outcome trials in patients with HFrEF confirmed benefits on HF outcomes for empagliflozin,^13^ dapagliflozin,^14^ and recently, sotagliflozin.^15^ Subgroup analyses indicate similar benefits for canagliflozin^16^ and ertugliflozin^17^; hence, salutary effects in HFrEF should be considered a class effect for SGLT2‐is. More importantly, these effects are consistently observed irrespective of the presence or absence of T2DM.^18^ Demonstrating a consistent reduction in CV mortality in HFrEF might require larger sample sizes as this effect was not present in the respective trials but a pooled meta‐analysis of *EMPEROR‐Reduced* and *DAPA‐HF* did indeed demonstrate significance.^19^

Recent imaging trials using CV magnetic resonance (CMR) to measure treatment effects of empagliflozin in HFrEF have reported positive effects on markers of cardiac remodelling.^20^, ^21^ Nevertheless, to the best of our knowledge, no study has assessed effects on cardiac energy metabolism in patients with HF and none of the previous studies evaluated the impact of empagliflozin in HFrEF and HFpEF separately.

Heart failure is associated with altered myocardial substrate metabolism and impaired myocardial energy production; hence, modulating cardiac energy metabolism may denote a promising future approach to treat HF.^22^, ^23^ The creatine kinase reaction delivers adenosine triphosphate (ATP) via phosphocreatine (PCr) from the mitochondria to the myofibrils in cardiomyocytes. In chronic HF, ATP demand outweighs its synthesis and PCr levels fall while ATP is reduced to a lesser extent. This results in a detectable depletion of the ratio of concentrations of PCr relative to ATP (PCr/ATP), which reflects the severity of energy starvation in the failing heart.^24^

The current study will evaluate whether treatment with empagliflozin leads to a measurable difference in PCr/ATP and thus to an increase in myocardial energy supply, which could improve resting and stress cardiac energetics, cardiac function, and exercise capacity. We aim to elucidate whether empagliflozin modulates cardiac energy metabolism, which in turn could contribute to the favourable effects observed in patients with HF.

## Study design

### Study design and study population

**Figure 1:**
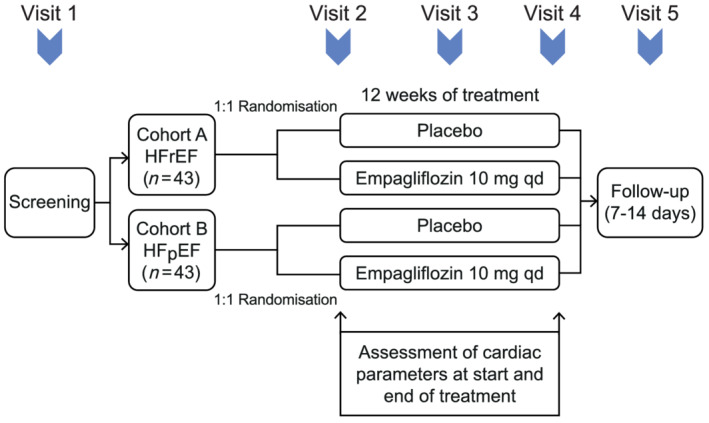
Schematic overview of the *EMPA‐VISION* study design. After screening (Visit 1; Day 0) and randomization, participants will be invited for dedicated assessment before randomization and treatment (Visit 2; Day 1). A safety assessment will be conducted after 2 weeks of treatment (Visit 3; Day 15 ± 1). Following treatment for 12 weeks, assessment will be repeated as described before (Visit 4; Day 84 ± 4). A final follow‐up will be carried out via telephone (Visit 5; Day 91 ± 7). HFpEF, heart failure with preserved ejection fraction; HFrEF, heart failure with reduced ejection fraction.

*EMPA‐VISION* is a randomized, double‐blind, controlled trial (*Figure* 1 and *Table* 1 for trial design and eligibility criteria, respectively) comparing the effects of empagliflozin and placebo on cardiac physiology and energy metabolism. Participants will be recruited into two cohorts consisting of up to 43 patients with HFrEF (Cohort A) and 43 patients with HFpEF (Cohort B), and, allowing for a maximum of 30% withdrawal rate, the minimum sample size will be 30 evaluable datasets per cohort. Participants will be randomized 1:1 via an interactive response technology system to receive either placebo or 10 mg of empagliflozin. Cardiac energy metabolism, cardiac structure, volumes and function as well as various biomarkers will be assessed before and after 12 weeks of treatment with empagliflozin.

**Table 1:** Simplified overview of the inclusion (including specific criteria for each of the two cohorts) and exclusion criteria of the *EMPA‐VISION* trial

Inclusion criteria	
• CHF ≥ 3 months • NYHA II–IV at screening • BMI < 40 kg/m^2^ • Age ≥ 18 years • Written informed consent	

Cohort A (HFrEF)	Cohort B (HFpEF)
• LVEF < 40% (measured by echocardiogram) • NT‐proBNP (>125 pg/mL) no AF • NT‐proBNP (>600 pg/mL) if diagnosed AF • Optimal medical therapy	• LVEF ≥ 50% • NT‐proBNP (>125 pg/mL) if free from AF • NT‐proBNP (>600 pg/mL) if diagnosed AF • LAVI > 34 mL/m^2^ • LVMI > 115 g/m^2^ (male) or >95 g/m^2^ (female) • Stable dose of oral diuretics > 1 week

Exclusion criteria
• Stroke or TIA < 6 months • Scars or non‐viable myocardium in the interventricular septum, unstable angina pectoris due to CAD, and major CV surgery (investigator opinion) • Any contraindication for CMR, CPET, and dobutamine stress test (including LVAD, ICD, CRT, or any cardiac device) • Heart transplant recipient or listed for heart transplant • Cardiomyopathy based on infiltrative diseases (e.g. amyloidosis), accumulation diseases (e.g. haemochromatosis and Fabry's disease), muscular dystrophies, cardiomyopathy with reversible causes (e.g. stress cardiomyopathy), hypertrophic obstructive cardiomyopathy, or known pericardial constriction • Moderate to severe uncorrected primary valvular heart disease • Acute decompensated HF (exacerbation of CHF) requiring i.v. diuretics, i.v. inotropes, or i.v. vasodilators, or LVAD or hospitalization • SBP ≥ 180 mmHg at screening. If SBP > 150 and <180 mmHg at screening on antihypertensive triple therapy • Symptomatic hypotension and/or an SBP < 100 mmHg at screening • Uncontrolled AF • Untreated ventricular arrhythmia with syncope documented within the 3 months prior to informed consent in patients without ICD • Diagnosis of cardiomyopathy induced by chemotherapy or peripartum <12 months prior to informed consent • Symptomatic bradycardia or second‐degree or third‐degree heart block in need of a pacemaker after adjusting beta‐blocker therapy or any other negative inotropic agents • Significant chronic pulmonary disease • Indication of liver disease, defined by serum levels of ALT, AST, or AP above 3 × upper limit of normal • Impaired renal function (creatinine clearance < 30 mL/min and/or dialysis) • Haemoglobin < 10 g/dL • T1DM • History of ketoacidosis • Major surgery <3 months prior or scheduled within trial • GI surgery or significant GI disorder • Active or suspected malignancy or history of malignancy within 2 years prior to informed consent • Any other disease than HF with a life expectancy of <1 year • Any drug considered likely to interfere with the safe conduct of the trial • Requirement for treatment with empagliflozin • Treatment with any SGLT2‐i or combined SGLT1‐i and SGLT2‐i • Currently enrolled in another investigational device or drug study, or <30 days between randomization and ending the other investigational trial • Known allergy or hypersensitivity to empagliflozin or other SGLT2‐is • Chronic alcohol or drug abuse • Women who are pregnant, breastfeeding, or who plan to become pregnant while in the trial

AF, atrial fibrillation; ALT, alanine aminotransferase; AP, alkaline phosphatase; AST, aspartate aminotransferase; BMI, body mass index; CAD, coronary artery disease; CHF, chronic heart failure; CMR, cardiovascular magnetic resonance; CPET, cardiopulmonary exercise testing; CRT, cardiac resynchronization therapy; CV, cardiovascular; GI, gastrointestinal; HF, heart failure; HFpEF, heart failure with preserved ejection fraction; HFrEF, heart failure with reduced ejection fraction; ICD, implantable cardioverter defibrillator; i.v., intravenous; LAVI, left atrial volume index; LVAD, left ventricular assist device; LVEF, left ventricular ejection fraction; LVMI, left ventricular mass index; NT‐proBNP, N‐terminal pro‐B‐type natriuretic peptide; NYHA, New York Heart Association; SBP, systolic blood pressure; SGLT1‐i, sodium‐glucose co‐transporter‐1 inhibitor; SGLT2‐i, sodium‐glucose co‐transporter‐2 inhibitor; T1DM, type 1 diabetes mellitus; TIA, transitory ischaemic attack.

### Study visits

*Figure* 1 provides a schematic overview of the *EMPA‐VISION* study visit schedule.

#### Visit 1—screening visit (Day 0)

After patients have provided informed consent, assessments are conducted at this visit to determine eligibility. These include echocardiogram (Echo), serum N‐terminal pro‐B‐type natriuretic peptide levels, multi‐slice coronary computed tomography angiography, a detailed physical exam, 12‐lead electrocardiogram, New York Heart Association classification, and blood sampling. Women of childbearing potential (WOCBP) undergo a pregnancy test, and pregnant women will be excluded from the trial. Eligible patients will proceed to Visit 2.

#### Visit 2—randomization and cardiovascular magnetic resonance assessment (Day 1)

**Figure 2:**
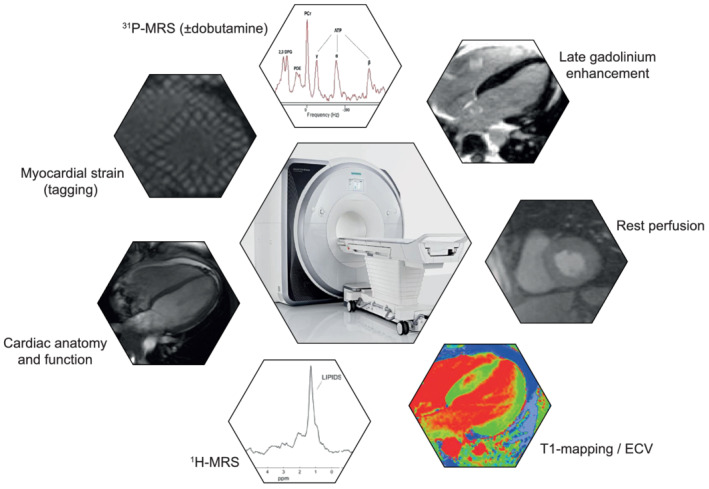
Overview of the cardiovascular magnetic resonance (CMR) techniques used in the *EMPA‐VISION* trial. All CMR sequences will be performed at a field strength of 3 Tesla (Siemens Healthineers, Erlangen, Germany). The CMR protocol is estimated to last approximately 2 h in total, split in two 1 h slots. After resting spectroscopy, dobutamine will be infused to target 65% of age maximal heart rate and stress spectroscopy will be acquired. ^1^H‐MRS, proton magnetic resonance spectroscopy; ^31^P‐MRS, phosphorus magnetic resonance spectroscopy; ECV, extracellular volume.

Participants are required to fast in preparation for the blood sampling and magnetic resonance spectroscopy (MRS) at this visit (no food or liquids except water for at least 6 h prior to sampling). Following confirmation of eligibility, participants will be randomized and allocated a treatment kit. Subsequently, Kansas City Cardiomyopathy Questionnaire (KCCQ) and five‐level EuroQol‐5D, blood sampling for various biomarkers and safety parameters, Echo, electrocardiogram, and body weight measurement will be repeated. Cardiopulmonary exercise testing (CPET) will be performed according to current recommendations.^25^ Furthermore, participants will perform a 6 min walking test (6MWT) according to the guidelines of the conducting centre. CMR including MRS in resting state and with dobutamine stress will be carried out as outlined in *Figure* 2. Patients will start the study medication after all baseline results are obtained. WOCBP undergo repeated pregnancy testing on site to ensure compliance with safety recommendations before starting the medication.

#### Visit 3—safety and compliance assessment (Day 15 ± 1)

The purpose of Visit 3 is to evaluate the participant's safety and confirm treatment adherence. The safety assessments include an Echo, urine testing for ketone body levels, body weight, vital signs, and a pregnancy test in WOCBP.

#### Visit 4—cardiovascular magnetic resonance assessment and end of treatment (Day 84 ± 4)

Representing the end of treatment, the assessments performed at Visit 2 are repeated following an overnight fast. All of the assessments must be performed within 48 h after participants have taken the last dose of their study medication. The final treatment adherence will be calculated before any excess medication is returned to pharmacy.

#### Visit 5—study completion (Day 91 ± 7)

Visit 5 marks the completion of the study for each individual participant. It is expected that this safety follow‐up will be performed via telephone although a clinic visit may be scheduled where required.

### Cardiovascular magnetic resonance

A schematic overview of *EMPA‐VISION's* CMR protocol is outlined in *Figure* 2. All acquisitions will be carried out using a field strength of 3 Tesla (Siemens Healthcare, Erlangen, Germany) under fasting conditions (i.e. absence of food for >6 h). The same MR scanner will be used for Visit 2 and Visit 4 assessments for each patient. Further information on CMR acquisition, details on resting and dobutamine stress MRS, and image and spectral analysis are provided in the Supporting Information section.

### Study endpoints

The primary endpoint of the *EMPA‐VISION* study is the change from baseline to Week 12 in PCr/ATP in the resting state measured by ^31^P‐MRS. An important exploratory endpoint is the difference from baseline to Week 12 in PCr/ATP under dobutamine stress, which may be more effective in identifying the mechanisms responsible for CV event prevention with empagliflozin. Other exploratory endpoints include changes in functional capacity (CPET and 6MWT), QoL questionnaires, cardiac structure and function, myocardial fat content, native T1‐mapping and extracellular volume, left atrial volume and emptying function, and metabolomics and biomarker assessments.

### Safety

There are no safety endpoints defined for this trial. However, starting after obtaining written informed consent, clinical and laboratory testing‐related adverse events (AEs) will be collected and coded using the Medical Dictionary for Drug Regulatory Activities, until a period of 7 days after the last dose of trial medication (residual effect period). All serious AEs and AEs of special interest will be assessed and promptly (within 24 h) reported to the sponsor. In addition, pre‐defined AEs of special interest such as hepatic injury, decrease in renal function, and ketoacidosis will be recorded.

### Ethical considerations

The study will be carried out in compliance with the ethical principles laid down in the Declaration of Helsinki, in accordance with the International Committee on Harmonisation's Guideline for Good Clinical Practice. The Clinical Trial Authorisation was obtained from the Medicines and Healthcare products Regulatory Agency, and ethical approval was granted by South Central Oxford C Research Ethics Committee (17/SC0262).

### Statistical considerations

To date, no data are available on the impact of SGLT2‐i treatment on PCr/ATP in patients with HF. Hence, sample size calculations in the current trial were based on previous results published by our own group,^26^ literature by other groups investigating augmentation of cardiac energy metabolism with perhexiline^27^ and trimetazidine,^28^ and expert advice. The study is designed to detect a difference for the primary endpoint (PCr/ATP) of 0.3 with a standard deviation of 0.28 achieving a power of 80%. The significance level for the sample size calculation is 0.05 two‐sided for each cohort. Taking into account the calculated effect size of 1.07, each cohort requires a minimum of 30 evaluable participants in order to achieve the aforementioned power level. Given the complex study protocol, we allowed for a maximum of 30% dropout rate, which would enable us to randomize up to 43 patients per cohort (86 patients in total) if needed.

## Discussion

*EMPA‐VISION* is the first randomized trial to assess cardiac energy metabolism in patients with HFrEF and HFpEF treated with empagliflozin.

### Why a mechanistic sodium‐glucose co‐transporter‐2 inhibitor trial?

The prime mechanism of action for many established HF medications remains elusive despite clear evidence for their efficacy. More importantly, none of the current pharmacological treatments shown to reduce mortality in HF were specifically developed to treat this devastating syndrome. This is equally true for SGLT2‐i where beneficial effects were initially discovered in subgroup analyses of CV safety trials for T2DM for empagliflozin (*EMPA‐REG OUTCOME*), dapagliflozin (*DECLARE‐TIMI 58*), and canagliflozin (*CANVAS*).^12^, ^29^, ^30^ The precise mechanisms of how SGLT2‐is exert their salubrious effects in patients with HF remain unclear and, even more importantly, might substantially differ in HFrEF and HFpEF, respectively. HF syndromes combine a broad variety of different aetiologies, but current drug treatment does not sufficiently reflect this underlying heterogeneity.^31^ Determining a mechanism of action for novel treatments is an important factor in predicting individual treatment response and improving overall treatment compliance.^32^ In addition, minimizing the time gap between diagnosis of HF and initiation of outcome‐improving treatment appears of particular importance for SGLT2‐i in the context of HFrEF.^33^ Sequaciously, the major strength of *EMPA‐VISION* is a comprehensive understanding of the effects of empagliflozin treatment on energy metabolism, imaging biomarkers, exercise capacity, and serum metabolomics. Results of this trial will stimulate research to investigate novel biological targets for future HF treatments and may potentially help improve phenotyping of patients with HF by identifying a subset of metabolic responders to SGLT2‐i treatment.

### Sodium‐glucose co‐transporter‐2 inhibitors and myocardial energy metabolism

The failing heart has previously been termed an ‘engine out of fuel’, and derangements in cardiac energy metabolism are considered a hallmark of cardiac failure.^23^, ^34^

Dedicated outcome trials in patients with HFrEF have confirmed a substantial risk reduction of HF events for empagliflozin, dapagliflozin, and sotagliflozin.^13^, ^14^, ^15^ In HFrEF, myocardial PCr/ATP is significantly reduced, correlates with CV mortality, and predicts prognosis more accurately than LVEF.^35^ Furthermore, the failing heart's ability to resynthesize ATP under increased workload is significantly blunted.^36^

Evidence for salutary effects of SGLT2‐i in patients with HFpEF is limited to smaller sample sizes but has been shown for empagliflozin, canagliflozin, and dapagliflozin.^37^, ^38^, ^39^ Additionally, subgroup analyses of *CANVAS (canagliflozin)* and *DECLARE (dapagliflozin)* provide larger‐scale evidence for a reduction of HF events in patients with HFpEF.^40^, ^41^ Patients with HFpEF exhibit similar reductions in myocardial energetics, and PCr/ATP correlates with the severity of diastolic dysfunction.^42^, ^43^ In small proof‐of‐principle studies, HF treatment and modulators of cardiac substrate metabolism improve PCr/ATP and cardiac function measurably within weeks,^28^, ^44^, ^45^ findings that have also been reproduced in randomized controlled trials.^46^, ^47^

**Figure 3:**
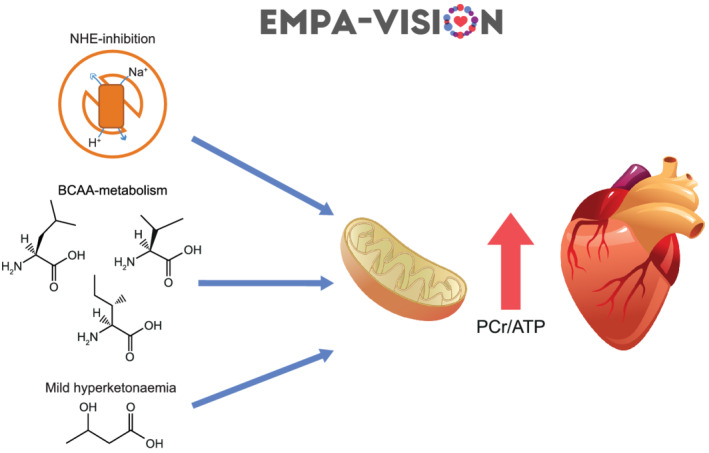
Postulated mechanisms by which empagliflozin might exert beneficial effects on patients with heart failure via manipulation of cardiac energy metabolism. Empagliflozin may enhance oxidative phosphorylation by inhibiting the cardiac isoform of the sodium/hydrogen‐exchanger 1 (NHE 1), promoting branched chain amino acid metabolism, and/or increasing ketone body oxidation. All of these effects would result in a measurable increase in cardiac energy production and storage, which in turn results in an increase in PCr/ATP as well as myocardial function. ATP, adenosine triphosphate; BCAA, branched chain amino acids; PCr, phosphocreatine.

Sodium‐glucose co‐transporter‐2 inhibitors have the potential to mitigate these derangements in cardiac energy metabolism,^48^ although this might be achieved via different pathways (*Figure* 3).

Ketone bodies provide an energy‐efficient fuel source for the failing heart, and the mild hyperketonaemia these drugs induce might thus be a reason for the effects observed with SGLT2‐is.^49^, ^50^, ^51^ Although evidence for this ‘super fuel’ hypothesis is conflicting and mainly generated from animal models, it warrants investigation as ketones have been suggested to preserve cardiac energetics under conditions of metabolic stress.^52^, ^53^, ^54^ Further studies investigating substrate metabolism in patients with HF suggest an increased breakdown of branched chain amino acids after treatment with empagliflozin, which could alleviate cardiac dysfunction.^55^, ^56^ However, impaired ATP production can also be ascribed to mitochondrial dysfunction,^57^ a process that SGLT2‐is seem to alleviate via inhibition of the sodium/hydrogen‐exchanger 1, which in turn would increase overall mitochondrial energy generation.^58^

*EMPA‐VISION's* additional serum metabolomics investigations add strength to our cardio‐metabolic assessments. The metabolome represents the totality of small metabolic intermediates of relevant biochemical pathways and with more than 40 000 molecules characterized already HFrEF and HFpEF each display a unique metabolic fingerprint.^59^, ^60^ Thus, metabolite biomarkers could assist clinical diagnosis, risk stratification, and identification of novel molecular targets.^61^ Not only will our metabolomic assessments help to subtype distinct metabolic profiles in patients with HF at baseline but also clarify whether enhancement of cardiac energy metabolism with SGLT2‐is might be translated differently in HFrEF and HFpEF, respectively.

Regardless of the underlying mechanisms, a sufficient amount of evidence suggests that SGLT2‐is might influence overall cardiomyocyte energy production, although the exact mechanisms remain to be elucidated. If empagliflozin increases myocardial energy production in patients with chronic HF, this should be detected by the comprehensive assessments in the *EMPA‐VISION* trial (*Figure* 3).

### Sodium‐glucose co‐transporter‐2 inhibitors, exercise physiology, and quality of life

Patients with HF experience a significant symptom burden that frequently leads to substantial reductions in QoL and exercise capacity.^62^ Modulating cardiac energy metabolism in HF has been shown to improve exercise capacity.^63^ Therefore, it is possible that SGLT2‐i treatment might lead to measurable differences in exercise capacity and perceived QoL. *EMPA‐VISION* will assess patient‐reported outcomes (KCCQ and five‐level EuroQol‐5D) and exercise capacity by means of CPET and 6MWT distance. Little is known about effects of SGLT2‐is on QoL and exercise capacity in HF. The *EMPERIAL* trials, investigating effects of empagliflozin treatment on exercise capacity and QoL in HFrEF and HFpEF, did not show a significant increase in 6MWT distance or QoL assessed by KCCQ.^64^ Equally, *DEFINE‐HF* (using dapagliflozin in patients with HFrEF) failed to demonstrate meaningful changes in 6MWT distance despite significantly improving QoL (KCCQ).^65^ It is worth noting that 6MWT distance was a secondary outcome in *DEFINE‐HF* but the ongoing *DETERMINE* trials (*NCT03877224*; *NCT03877237*) will investigate effects of dapagliflozin treatment on 6MWT distance and QoL as co‐primary endpoints in HFrEF and HFpEF. Of note, secondary analyses of *EMPEROR‐Reduced* and *DAPA‐HF* suggest a sustained improvement in KCCQ.^66^, ^67^

The 6 min walking tests entail a few limitations as they are highly effort dependent, are potentially falsely negative due to non‐cardiac reasons (e.g. injuries), and do not correlate specifically well with QoL.^68^ A more reliable and accurate test to investigate exercise capacity and cardio‐respiratory fitness is CPET, which correlates well with both QoL and metabolic dysfunction.^69^ More importantly, trials using empagliflozin in patients with HFrEF suggest positive effects on cardio‐respiratory fitness determined by CPET.^21^, ^70^ Thus, although these exercise test results in our *EMPA‐VISION* trial are considered exploratory, we are confident they will augment our CMR findings and provide deeper understanding on metabolic modulation by SGLT2‐is in HF.

## Conclusions

*EMPA‐VISION* is the first randomized controlled trial to investigate effects of empagliflozin treatment on cardiac energy metabolism and cardiac physiology in patients with HFrEF and HFpEF, with or without T2DM. It is designed to provide a possible explanation for the benefits observed in CV outcome trials with SGLT2‐is such as *EMPA‐REG OUTCOME*,^12^ *CANVAS*,^29^ and *DECLARE*.^30^ In addition, it may provide a mechanism of action for HF outcomes in *DAPA‐HF* ^14^ and *EMPEROR‐Reduced*,^13^ the ongoing *EMPEROR‐Preserved*,^71^ and *DELIVER* (*NCT03619213*) trials and further insights into results of the *EMPERIAL* exercise capacity and symptom burden trials.^64^ Consequently, our trial may promote development of targeted treatments to modulate high‐energy phosphate metabolism in the failing heart and stimulate further research on SGLT2‐is and cardiac energy metabolism.

## Conflict of interest

M.H., M.M., and S.N. are supported by an industrial grant provided by Boehringer Ingelheim. R.G. and H.N. are employees of Boehringer Ingelheim Pharma GmbH & Co. KG. The views expressed are those of the author(s) and not necessarily those of the NHS, the NIHR, or the Department of Health.

## Funding

Boehringer Ingelheim is the sponsor of the *EMPA‐VISION* study and was involved in its study design. Boehringer Ingelheim also supported the preparation of this article. S.N. acknowledges support from the Oxford British Heart Foundation (BHF) Centre of Research Excellence. R.R.H. and S.N. were supported by the Oxford National Institute for Health Research (NIHR) Biomedical Research Centre (BRC). C.T.R. and L.V. are funded by a Sir Henry Dale Fellowship from the Wellcome Trust and the Royal Society (098436/Z/12/B and 221805/Z/20/Z, respectively). L.V. also gratefully acknowledges support of the Slovak Grant Agencies VEGA (2/0001/17) and APVV (15‐0029).

## Supporting information


**Data S1.** Supporting information.
