# Supplementary material for: Design and rationale of the EMPA‐VISION trial: investigating the metabolic effects of empagliflozin in patients with heart failure
Source: ESC Heart Fail. 2021 May 6;8(4):2580–90. doi: 10.1002/ehf2.13406 (PMC8318430; doi:10.1002/ehf2.13406)
Supplement: Supplementary file 1 — Data S1. Supporting information. [file EHF2-8-2580-s001.docx]

**Design and rationale of the EMPA-VISION trial: investigating the metabolic effects of empagliflozin in heart failure patients**

Supporting Information for ESCHF-20-01006

**Magnetic Resonance Spectroscopy**

All magnetic resonance spectroscopy (MRS) will be done under fasting conditions for all participants. Prior to undergoing MRS, the patient will be cannulated to allow the administration of dobutamine while in the scanner.
Phosphorus spectra are obtained using a 3T MR system (TRIO; Siemens Healthcare, Erlangen, Germany). The patient is positioned prone. A three-element radiofrequency (RF) surface coil is used for PCr/ATP estimation. One element (28 x 26 cm) is dual tuned to 1H (127.7MHz) and 31P (49.8MHz) and acts as a local 1H transmit-receive and 31P transmit coil. 31P signal is received by the loop (12 x 15 cm) and butterfly (23 x 12 cm) elements of the coil. Lipid spectra are obtained in supine position using a 3T MR system (PRISMA; Siemens Healthcare, Erlangen, Germany). A 24- channel spinal matrix coil is placed beneath the subject while a 18-channel body matrix coil is placed over the anatomical region of interest.

**31 P-MRS**

After the participant is positioned prone over the centre of the coil and moved to the isocentre of the MR scanner, FID inversion-recovery data (inversion delay 100-3000ms) of phenylphosphonic acid (PPA) fiducial placed in the RF coil, used to calculate coil loading, and FLASH images of cod liver oil capsules also placed in the coil, used to determine coil position and rotation, are acquired together with a 3D-stack of short axis localizers. Next, three saturation bands are positioned over liver and skeletal muscle while the middle of the 3D chemical shift imaging (CSI) acquisition matrix (8×16×8 over a 240×240×200 mm^3^ field of view) is placed over the basal-mid interventricular septum of the left ventricle. Our non-gated, 3D acquisition-weighted, ultra-short TE CSI (UTE-CSI) sequence takes approximately 11 minutes.(1) The centre frequency is set -250 Hz from PCr, i.e. between γ- and α-ATP, so that the excitation bandwith covers the whole spectrum, from β-ATP on the one side to phospho-monoesters on the other.

After resting spectroscopy, dobutamine will be infused intravenously at incremental rates between 5 and 40 μg/kg with a target of 65% of age-maximal heart rate. During this time, blood pressure will be measured every 2-3 minutes. Heart rate, pulse oximetry, and 3-channel ECG will also be monitored continuously during dobutamine infusion. Heart rate will be maintained at target for the duration of the acquisition.(2) Where possible, additional cine imaging will be acquired at rest and during dobutamine stress for assessment of cardiac function.

Selecting the mid septal voxel, OXSA fitting toolbox(3) is used to fit the raw time-domain signal with the AMARES algorithm, taking into account prior knowledge (expected frequency shifts, j-coupling constants for ATP and linewidths constrained to ±20 Hz around the linewidth of PCr. Fitted data are corrected for partial saturation using metabolite-specific Tl values and experimental acquisition parameters, e.g., TR and flip-angle calculated for the selected voxel using Biot Savart Law distribution from the field within the coil calculated from the IR FIDs and coil position data. In addition, the PCr/ATP ratio is corrected for blood contamination according to (CITE)

**1 H-MRS**

After correct patient positioning and acquisition of anatomical images, cardiac spectra are obtained from the mid-interventricular septum using a stimulated echo sequence (STEAM).(4). Spectroscopic acquisitions are performed using ECG-trigger at end-diastole and in expiration to minimize motion artefacts. Water suppressed data are acquired over 5 breath holds, acompassing 5 acquisitions each. Water unsuppressed spectra are then acquired (for internal reference) within one breath hold.

Spectral quantification is again performed in the time domain, using the AMARES algorithm included in the OXSA toolbox. The amplitude of the lipid resonance at 1.3 ppm (-CH_2_-) from the spectra is selected for myocardial lipid quantification. The lipid content is calculated as a percentage relative to water as the amplitude of the lipid peak divided by the amplitude of the water peak, and multiplied by 100.

**Cardiac Magnetic Resonance Imaging**

Cardiac magnetic resonance (CMR) imaging will be performed on a 3 Tesla MR scanner (PRISMA; Siemens Healthcare, Erlangen, Germany). The same scanner will be used for the Visit 2 and Visit 4 assessment for each patient. Prior to undergoing CMR, the patient will be cannulated to allow the administration of contrast agent while in the scanner. All imaging will be done under fasting conditions for all participants. Patients will lie in a supine position and a dedicated 18-channel cardiac coil will be placed around the patient’s chest together with a 24-channel spinal matrix coil underneath them. Images will be obtained using breath hold and ECG-gating.

**Cardiac Volumes and Function**

Cine images will be acquired for cardiac volumes, mass and function using steady state free precession cine imaging as recommended.(5) Cardiac volumes are acquired using steady state free precession (SSFP) imaging. Scan parameters are typically: voxel size 2.0x2.0x8.0mm, field of view=380x380mm, TR/TE 39.6/1.12ms, flip angle 55◦, matrix 192x192, GRAPPA=3, 24 reference lines, segments=15, concatenations=1). Pilot images are initially acquired and used to plan and acquire horizontal long axis (HLA), vertical long axis (VLA), left ventricular outflow tract (LVOT) long axis and short-axis stack images. Retrospectively-gated acquisition is the default and used for all patients in sinus rhythm at the time of scanning. The acquisition method for patients in AF at the time of scanning are prospectively triggered image acquisitions when acceptable image quality cannot be obtained. LV short axis epicardial and endocardial borders are manually contoured at end diastole and end systole. LVESV and LVEDV are used to calculate SV (SV = EDV-ESV). LVEF and cardiac output (CO) are calculated (EF = SV/EDV, CO=SV x HR). LV mass is also calculated by subtracting the endocardial volume from the epicardial volume, based on prior knowledge of myocardial specific gravity (1.05 g/cm3).

**Tagging**

Tagged MR images will be acquired before administration of gadolinium for measurement of LV-strain, using an ECG-triggered segmented k-space fast gradient echo sequence with spatial modulation of magnetization in orthogonal planes creating a square grid of parallel tag lines.(6) Three short axis (basal, mid and apical slice respectively) and one long axis (horizontal) images are obtained. The scan parameters are typically: voxel size 2.1 x 1.4 x 8.0 mm, field of view = 360 x 292 mm, matrix 141 x 256, TR/TE = 40.45/3.89 ms, flip angle 14◦, segments = 9, phases = 16, concatenations = 1, grid tag distance = 7mm, bandwidth = 184 Hz/Px. Post-processing analysis is performed using CIM software (CIMTag2D, Auckland, New Zealand). Semi-automated analysis is performed by aligning a grid to the myocardial tagging planes in end-diastole. End-systole is determined visually, and tags adjusted at each frame through the cardiac cycle to derive peak systolic circumferential strain for the mid-ventricular slice, which is expressed as a percentage change from end-diastole. Normal strain has previously been described as -19 ± 234; impaired myocardial contractility is shown by a more positive value.

**T1-Mapping**

T1-mapping and extracellular volume quantification (ECV) will be performed using a shortened modified look- locker inversion recovery (ShMOLLI) sequence as previously described.(7) Native T1-mapping (i.e. pre-contrast acquisition) will be performed on one mid-ventricular short-axis slice and repeated twice in the same location. Post-contrast T1-mapping will be repeated 15 minutes after administration of contrast agent in the identical mid- ventricular short-axis slice position as before. Briefly, T1-maps are based on 5-7 images with specific TI = 100-5000 ms, collected using SSFP readouts in a single breath-hold, typically: TR/TE = 201.32/1.07 ms, flip angle = 35◦, FOV = 340x255 mm, matrix = 192x144, 107 phase encoding steps, interpolated voxel size = 0.9 x 0.9 x 8 mm, GRAPPA = 2, 24 reference lines, cardiac delay time TD = 0 ms or 500 ms (see below); 206 ms acquisition time for single image, phase partial Fourier 6/8, segments = 79, measurements = 1, bandwidth = 898 Hz/Px. To assess for how well T1 model fitting is achieved for each T1-map, parametric maps of the goodness of fit (R2-maps) are used. R2-maps provide an additional means to identify areas with potentially compromised T1 accuracy and allow critical assessment of the quality of T1-maps generated.(8) Offline post-processing involves manual tracing of endocardial and epicardial contours for T1-measurements in myocardial segments 1 to 16 of the American Heart Association (AHA) 17-segment model using dedicated in-house MC-ROI software (programmed in Interactive Data Language v6.1 by Prof. Stefan Piechnik).(9)

**Resting Perfusion**

A five measurement test scan without contrast agent is run first and any problems identified (e.g. artefacts, breath- holding or ECG-mistriggering) are addressed. Perfusion imaging is performed every cardiac cycle during the first pass of intravenous MR contrast agent (0.1mmol/kg body weight of Gadobutrol; Gadovist®, Bayer Pharmaceutical, Reading, United Kingdom) using a T1-weighted fast (spoiled) gradient echo sequence on three short axis cine images. Scan parameters are typically: voxel size 2.8 x 2.3 x 10.0 mm, field of view = 360 x 270 mm, matrix 96 x 160, TR/TE = 160.76/1.05 ms, flip angle 12◦, TI 100 ms, GRAPPA = 2, 18 reference lines, measurements = 50, segments = 57, concatenations = 1, phases = 1, 1 shot per slice, bandwidth 651 Hz/Px. Number of measurements is usually between 60 and 75 but may be adjusted for very low cardiac output in HF patients.

**Late Gadolinium Imaging**

Late gadolinium enhancement (LGE) will be performed with an inversion-recovery-prepared, segmented gradient echo sequence after a 6-10 minute time delay following administration of a gadolinium-based contrast agent (0.1mmol/kg body weight of Gadobutrol; Gadovist®, Bayer Pharmaceutical, Reading, UK) for resting perfusion and provides quantification of regional fibrotic tissue. The inversion time is adjusted for optimal nulling of remote normal myocardium and is assessed visually by the scan operator. The scan parameters are typically: voxel size 2.0 x 1.5 x 8.0 mm, matrix 144x256, field of view = 380x285mm, TR/TE = 800.20/3.36ms, flip angle 25◦ GRAPPA = 2, 24 reference lines, segments = 25, phases = 1, concatenations = 1, measurements = 1, bandwidth = 130Hz/Px).

1. Tyler DJ, Emmanuel Y, Cochlin LE, Hudsmith LE, Holloway CJ, Neubauer S, Clarke K, Robson MD. Reproducibility of 31P cardiac magnetic resonance spectroscopy at 3 T. *Nmr Biomed*. 2009 May;**22**(4):405-413.

2. Rider OJ, Francis JM, Ali MK, Holloway C, Pegg T, Robson MD, Tyler D, Byrne J, Clarke K, Neubauer S. Effects of catecholamine stress on diastolic function and myocardial energetics in obesity. *Circulation*. 2012 Mar 27;**125**(12):1511-1519.

3. Purvis LAB, Clarke WT, Valkovic L, Levick C, Pavlides M, Barnes E, Cobbold JF, Robson MD, Rodgers CT. Phosphodiester content measured in human liver by in vivo (31) P MR spectroscopy at 7 tesla. *Magn Reson Med*. 2017 Dec;**78**(6):2095-2105.

4. Rial B, Robson MD, Neubauer S, Schneider JE. Rapid quantification of myocardial lipid content in humans using single breath-hold 1H MRS at 3 Tesla. *Magn Reson Med*. 2011 Sep;**66**(3):619-624.

5. Hudsmith LE, Petersen SE, Francis JM, Robson MD, Neubauer S. Normal human left and right ventricular and left atrial dimensions using steady state free precession magnetic resonance imaging. *J Cardiovasc Magn Reson*. 2005;**7**(5):775-782.

6. Stuber M, Spiegel MA, Fischer SE, Scheidegger MB, Danias PG, Pedersen EM, Boesiger P. Single breath-hold slice-following CSPAMM myocardial tagging. *MAGMA*. 1999 Oct;**9**(1-2):85-91.

7. Piechnik SK, Ferreira VM, Dall'Armellina E, Cochlin LE, Greiser A, Neubauer S, Robson MD. Shortened Modified Look-Locker Inversion recovery (ShMOLLI) for clinical myocardial T1-mapping at 1.5 and 3 T within a 9 heartbeat breathhold. *J Cardiovasc Magn Reson*. 2010 Nov 19;**12**:69.

8. Ferreira VM, Piechnik SK, Dall'Armellina E, Karamitsos TD, Francis JM, Choudhury RP, Friedrich MG, Robson MD, Neubauer S. Non-contrast T1-mapping detects acute myocardial edema with high diagnostic accuracy: a comparison to T2-weighted cardiovascular magnetic resonance. *J Cardiovasc Magn Reson*. 2012 Jun 21;**14**:42.

9. Cerqueira MD, Weissman NJ, Dilsizian V, Jacobs AK, Kaul S, Laskey WK, Pennell DJ, Rumberger JA, Ryan T, Verani MS, American Heart Association Writing Group on Myocardial S, Registration for Cardiac I. Standardized myocardial segmentation and nomenclature for tomographic imaging of the heart. A statement for healthcare professionals from the Cardiac Imaging Committee of the Council on Clinical Cardiology of the American Heart Association. *Circulation*. 2002 Jan 29;**105**(4):539-542.
